# Molecular Features Underlying Selectivity in Chicken Bitter Taste Receptors

**DOI:** 10.3389/fmolb.2018.00006

**Published:** 2018-01-31

**Authors:** Antonella Di Pizio, Nitzan Shy, Maik Behrens, Wolfgang Meyerhof, Masha Y. Niv

**Affiliations:** ^1^The Robert H Smith Faculty of Agriculture, Food and Environment, The Institute of Biochemistry, Food and Nutrition, The Hebrew University, Rehovot, Israel; ^2^The Fritz Haber Center for Molecular Dynamics, The Hebrew University, Jerusalem, Israel; ^3^Department of Molecular Genetics, German Institute of Human Nutrition Potsdam-Rehbruecke, Nuthetal, Germany; ^4^Center for Integrative Physiology and Molecular Medicine, Saarland University, Homburg, Germany

**Keywords:** bitter compounds, chicken Tas2rs, GPCRs, homology modeling, induced-fit docking, virtual screening, calcium-mobilization assays

## Abstract

Chickens sense the bitter taste of structurally different molecules with merely three bitter taste receptors (*Gallus gallus* taste 2 receptors, ggTas2rs), representing a minimal case of bitter perception. Some bitter compounds like quinine, diphenidol and chlorpheniramine, activate all three ggTas2rs, while others selectively activate one or two of the receptors. We focus on bitter compounds with different selectivity profiles toward the three receptors, to shed light on the molecular recognition complexity in bitter taste. Using homology modeling and induced-fit docking simulations, we investigated the binding modes of ggTas2r agonists. Interestingly, promiscuous compounds are predicted to establish polar interactions with position 6.51 and hydrophobic interactions with positions 3.32 and 5.42 in all ggTas2rs; whereas certain residues are responsible for receptor selectivity. Lys^3.29^ and Asn^3.36^ are suggested as ggTas2r1-specificity-conferring residues; Gln^6.55^ as ggTas2r2-specificity-conferring residue; Ser^5.38^ and Gln^7.42^ as ggTas2r7-specificity conferring residues. The selectivity profile of quinine analogs, quinidine, epiquinidine and ethylhydrocupreine, was then characterized by combining calcium-imaging experiments and *in silico* approaches. ggTas2r models were used to virtually screen BitterDB compounds. ~50% of compounds known to be bitter to human are likely to be bitter to chicken, with 25, 20, 37% predicted to be ggTas2r1, ggTas2r2, ggTas2r7 agonists, respectively. Predicted ggTas2rs agonists can be tested with *in vitro* and *in vivo* experiments, contributing to our understanding of bitter taste in chicken and, consequently, to the improvement of chicken feed.

## Introduction

Bitter taste is one of the basic taste modalities thought to protect organisms from consuming poisons that are often bitter. Bitter taste perception is mediated by bitter taste receptors (Tas2rs), a subfamily of Class A G-protein coupled receptors (GPCRs) (Di Pizio and Niv, 2014; Di Pizio et al., 2016). Recently, it has been shown that Tas2rs are expressed in tissues other than the mouth (Behrens and Meyerhof, 2010; Clark et al., 2012). These extra-oral Tas2rs have been implicated in diverse functions, including cellular responses to toxins (Lee and Cohen, 2014), bronchodilation (Ting-A-Kee et al., 2015), and regulation of thyroid hormones (Clark et al., 2015), suggesting that sensing bitter compounds is likely to have physiological roles beyond food evaluation.

The molecular recognition of bitter molecules by their receptors is rather complex. The structures of close to 700 bitter compounds have been gathered in the BitterDB database (http://bitterdb.agri.huji.ac.il; Wiener et al., 2012), which currently contains a special session dedicated to chicken (http://bitterdb.agri.huji.ac.il/dbbitter.php?mode_organism=Chicken). However, the number of bitter compounds is estimated to be thousands (Meyerhof et al., 2010) as recently confirmed by the BitterPredict protocol (Dagan-Wiener et al., 2017). In humans, these numerous compounds are perceived as bitter by 25 receptors (TAS2Rs); some of the receptors are still orphan, or have few known agonists, while others can be activated by numerous and structurally dissimilar compounds (Di Pizio and Niv, 2015). Similarly, the ligands vary in the repertoire of bitter receptors that they activate: some bitter compounds are selective toward a single TAS2R, while others activate multiple TAS2Rs (Di Pizio and Niv, 2015).

Interestingly, the number of Tas2rs varies by species. Chickens sense the bitter taste with merely three chicken (*Gallus gallus*) bitter taste receptors, ggTas2r1, ggTas2r2, ggTas2r7 (Go, 2006), which are expressed, together with their downstream signaling components, in both gustatory and extra-gustatory tissues (Cheled-Shoval et al., 2014, 2015). With their small Tas2r repertoire, chickens represent an optimal system to investigate the molecular mechanism of bitter taste perception. In 2014, a set of 46 bitter compounds, previously screened on human bitter taste receptors (Meyerhof et al., 2010), has been profiled against all chicken bitter taste receptors under the same assay conditions (Behrens et al., 2014), leading to the de-orphanization of ggTas2rs and furnishing a consistent dataset of ggTas2r agonists. The results of this screening indeed demonstrated that the smaller repertoire of ggTas2rs compared to human TAS2Rs is partially compensated by a broader repertoire of ligands. Even though ggTas2rs appear to be promiscuous and tend to accommodate many chemically diverse compounds, bitter compounds may exhibit different selectivity/promiscuity profiles toward chicken bitter taste receptors, and can be very selective for a specific subtype (Behrens et al., 2014). So far, a total of 25 bitter agonists for the three chicken Tas2rs and an antagonist for ggTas2r1 and ggTas2r7 have been unraveled (Behrens et al., 2014; Hirose et al., 2015; Dey et al., 2016; Cheled-Shoval et al., 2017). We took advantage of the simple ggTas2r system to compare *in vitro* vs. *in vivo* detection thresholds of selective and promiscuous ggTas2r agonists. In general, *in vivo* thresholds were similar or up to two orders of magnitude higher than the *in vitro* ones, but the *in vivo*:*in vitro* ratios were different for different ligands and ggTas2r-promiscuous ligands did not exhibit lower ratios than ggTas2r-selective ligands (Cheled-Shoval et al., 2017). Recently, integrating *in silico* and *in vitro* experiments on ggTas2r1 we investigated the binding modes of known agonists into the binding site and predicted additional ligands, providing a docking strategy for chemosensory receptors and other GPCRs, where the sequence identity between models and templates is very low (Di Pizio et al., 2017). Here, we use a similar approach to analyze the promiscuity/selectivity profile of bitter compounds in chicken, aiming to unravel what makes compounds active toward all ggTas2rs or selective for a particular subtype.

## Results and discussion

### Tas2r-ligand relations

**Figure 1:**
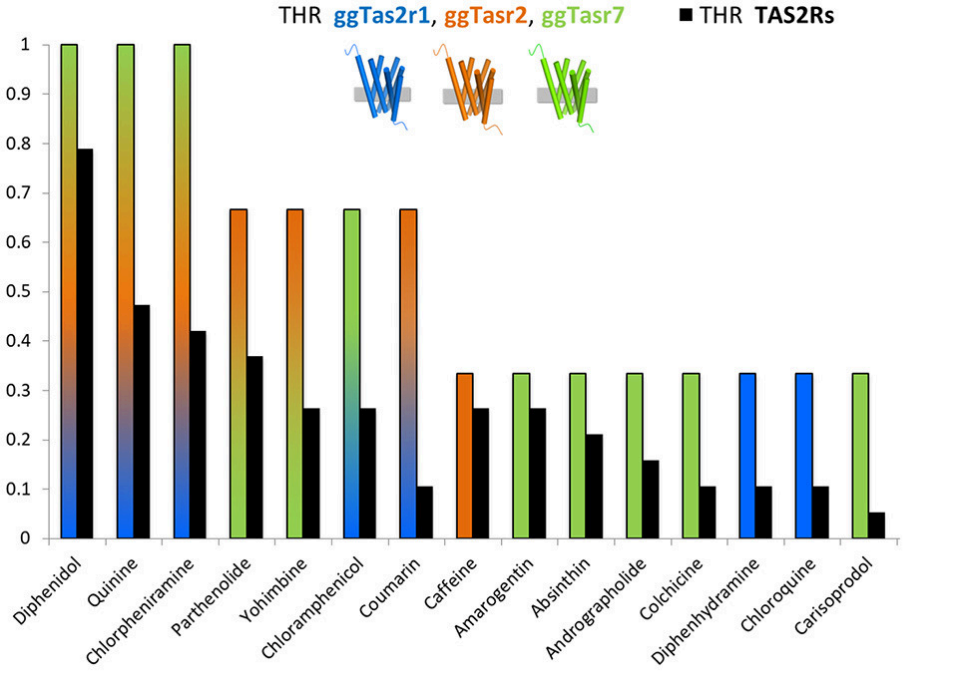
THR (target hit-rate) of bitter compounds toward human TAS2Rs (black bars) and chicken ggTas2r1, ggTas2r2, ggTas2r7 (blue, orange, and green bars, respectively). THR parameter is the number of targets hit at a specific concentration divided by the number of targets tested (Azzaoui et al., 2007; Di Pizio and Niv, 2015). Specifically, only compounds that elicited receptor activation of both human TAS2Rs and ggTas2rs at concentration of 300 μM or lower were used to generate this graph.

Figure 1 represents the ligand repertoire of ggTas2rs vs. that of TAS2Rs. Promiscuous compounds for chicken activate several human TAS2Rs, and the most selective compounds for chicken are selective for humans as well. Therefore, understanding how selectivity is achieved in chicken may provide insights about the selectivity of bitter compounds in humans.

Among the compounds reported in Figure 1, we can observe ggTas2r1-selective molecules, i.e., diphenhydramine and chloroquine, ggTas2r2-selective molecules, i.e., caffeine, ggTas2r7-selective compounds, i.e., amarogentin, andrographolide, etc.; but also promiscuous compounds - diphenidol, quinine and chlorpheniramine activate all ggTas2rs; or ligands with an intermediate promiscuity toward the chicken receptors - parthenolide and yohimbine activate ggTas2r2 and ggTas2r7, while coumarin activate ggTas2r1 and ggTas2r2, and chloramphenicol responds to ggTas2r1 and ggTas2r7.

### Binding pocket of ggTas2rs

In order to identify the specific residues that may be responsible for the selectivity of each ggTas2r toward their agonists, we analyzed similarities and differences in the binding site. As previous works on human bitter taste receptors suggested (Brockhoff et al., 2010; Born et al., 2013; Karaman et al., 2016), the location of the binding site in ggTas2r1 coincides with the canonical binding site of Class A GPCRs (Di Pizio et al., 2017). Importantly, our recent investigation of the ggTas2r1 agonist-bound conformation led to identify Lys86^3.29^, Phe89^3.32^, Asn93^3.36^, Phe181^5.38^, Leu185^5.42^, Tyr244^6.47^, Asn247^6.51^, and Leu251^6.55^ as the ggTas2r1 residues involved in agonist binding and recognition (Di Pizio et al., 2017). Transmembrane (TM) residues are identified throughout the text by a superscript numbering system according to the Ballesteros-Weinstein (BW) numbering method, where the residue corresponding to the Class A GPCRs most conserved residue in TM number X is assigned the index X.50, and the remaining residues are numbered relative to this position (Ballesteros and Weinstein, 1995).

Table 1 shows the different compositions of the agonist-interacting residues among the three chicken bitter taste receptors. Only three residues are conserved between the receptors ggTas2r1 and ggTas2r2 (Asn^3.36^, Leu^5.42^, Tyr^6.47^), one residue is conserved between ggTas2r1 and ggTas2r7 (Leu^6.55^), while no residues are conserved between ggTas2r2 and ggTas2r7. ggTas2r7 differs from the other two receptors: indeed, in the phylogenetic tree in the Figure 1 of the paper from Behrens et al. (2014), ggTas2r1 and ggTas2r2 are grouped together, while ggTas2r7 appears in a separate branch. Interestingly, Asn^3.36^ and Asn^6.51^, found to be relevant in agonist/ggTas2r1 binding (Di Pizio et al., 2017), are replaced by Asn^3.36^-Ser^6.51^, and Ser^3.36^-His^6.51^ combinations in ggTas2r2 and ggTas2r7, respectively. Phe^3.32^ and Phe^5.38^, involved in aromatic interactions in agonist-ggTas2r1 complexes (Di Pizio et al., 2017), are replaced by Trp^3.32^ and Tyr^5.38^, and Met^3.32^ and Ser^5.38^ in ggTas2r2 and ggTas2r7, respectively.

**Table 1:** Agonist-interacting residues in ggTas2rs.

**BW**	**ggTas2r1**	**ggTas2r2**	**ggTas2r7**	TAS2Rs^*^
3.29	Lys86	Gly85	Ala91	Not-conserved
3.32	Phe89	Trp88	Met94	Trp/Phe
3.36	Asn93	Asn92	Ser98	Asn
5.38	Phe181	Tyr180	Ser186	Not-conserved
5.42	Leu185	Leu184	Ile190	Hydrophobic
6.47	Tyr244	Tyr243	Phe247	Not-conserved
6.51	Asn247	Ser246	His250	Polar residue
6.55	Leu251	Gln250	Leu254	Not-conserved

* *For comparison, this column shows if analyzed residues are conserved or not in human TAS2Rs*.

*Cells are colored by the same color for residues that are conserved among bitter taste receptors*.

### Binding modes of promiscuous vs. selective compounds

To investigate the influence of amino acid differences on the binding and the selectivity profile of bitter compounds, we analyzed the binding modes of both selective and promiscuous ligands in the cognate receptors. We built ggTas2r2 and ggTas2r7 models, analyzed the binding modes of their agonists with induced-fit docking simulations, and compared the previously predicted agonist/ggTas2r1 binding poses (Di Pizio et al., 2017) with those analyzed here. A matrix with all interactions found for each agonist/ggTas2r complex is reported in Supplementary Figure S1.

**Figure 2:**
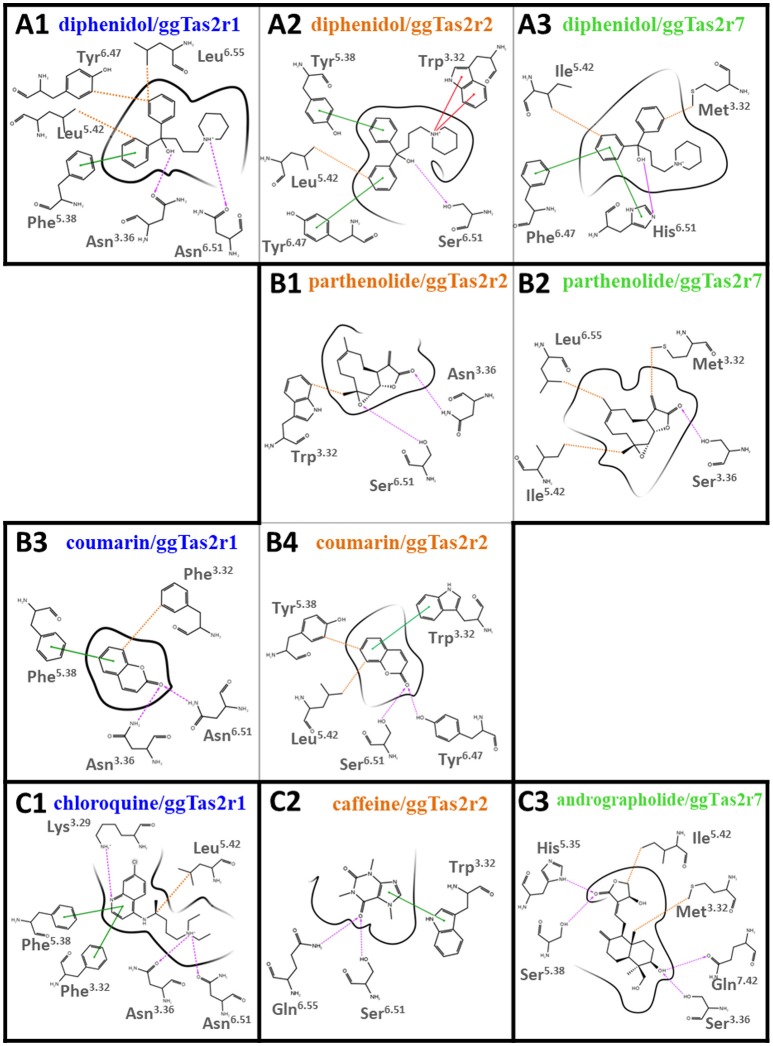
2D representation of predicted binding modes of promiscuous **(A)**, intermediate-promiscuous **(B)** and selective compounds **(C)** into the ggTas2r binding sites. H-bond interactions are shown as magenta dashed arrows, π-π interactions as green lines, cation-π interactions as red lines and hydrophobic interactions are represented as the distances between the closest heavy atoms of the ligand and the residue as orange dotted lines. Binding modes of diphenidol, coumarin, and chloroquine into ggTas2r1 have been described in our previous work (Di Pizio et al., 2017).

*Binding modes of promiscuous compounds*, e.g., diphenidol (Figure 2A and Supplementary Figure S2A): diphenidol in complex with ggTas2r1 forms H-bonds with Asn^3.36^ and Asn^6.51^, π-π stacking interaction with Phe^5.38^, and hydrophobic interactions with Leu^5.42^, Tyr^6.47^, and with Phe^3.32^ (Figure 2A1; Di Pizio et al., 2017). These interactions are well-conserved in diphenidol/ggTas2r2 and diphenidol/ggTas2r7 complexes (Figures 2A2,A3). In all cases we observe an H-bond interaction in position 6.51 (Ser in ggTas2r2 and His in ggTas2r7), and hydrophobic or aromatic interactions in positions 3.32, 5.42, 6.47. This allows diphenidol to assume a similar conformation in all three receptors with only slight differences: the π-π interaction with Phe^5.38^ in ggTas2r1 is replaced by a π-π stacking interaction with Tyr^5.38^ in ggTas2r2, but is lacking in ggTas2r7 where we have a polar residue, i.e., Ser, in this position; the H-bond between the ligand and ggTas2r1 Asn^3.36^ is not conserved in the other two receptors.

*Binding modes of intermediate-promiscuous compounds*, e.g., parthenolide and coumarin (Figure 2B and Supplementary Figure S2B). Parthenolide activates ggTas2r2 and ggTas2r7: it forms H-bonds with Asn^3.36^ and Ser^6.51^, and hydrophobic interactions with Trp^3.32^ when in complex with ggTas2r2 (Figure 2B1); similarly, it forms an H-bond with Ser^3.36^ and hydrophobic interactions with Met^3.32^, Ile^5.42^, and Leu^6.55^ in the ggTas2r7 binding site (Figure 2B2). Coumarin activates ggTas2r1 and ggTas2r2: in complex with ggTas2r1, it establishes H-bonds with Asn^3.36^ and Asn^6.51^, π-π stacking interaction with Phe^5.38^, and hydrophobic interactions with Phe^3.32^ (Figure 2B3; Di Pizio et al., 2017). The H-bond interaction between the ligand and the residue in position 6.51 is conserved in the ggTas2r2 complex as well, the H-bond in position 3.36 is lacking and replaced by an additional H-bond with Tyr^6.47^ (Figure 2B4). Moreover, in coumarin/ggTas2r2 binding pose, hydrophobic and π-π stacking interactions are observed with Leu^5.42^ and Tyr^5.38^, and Trp^3.32^, respectively (Figure 2B4).

*Binding modes of selective compounds*, e.g., chloroquine, caffeine, and andrographolide (Figure 2C and Supplementary Figure S2C). Chloroquine is selective for ggTas2r1 and binds Lys^3.29^, Asn^3.36^, and Asn^6.51^ through H-bonds, Phe^3.32^ and Phe^5.38^ through π-π stacking interactions, Leu^5.42^ with hydrophobic interactions. Lys^3.29^ is replaced by a glycine and an alanine in ggTas2r2 and ggTas2r7, respectively, and may be responsible for the selectivity of chloroquine for ggTas2r1. Indeed, it has been shown that mutating Lys^3.29^ to Ala causes severe impairments of the ggTas2r1 activation by chloroquine (Di Pizio et al., 2017). Caffeine is selective for ggTas2r2 and interacts with Trp^3.32^ through π-π stacking interaction, and with Ser^6.51^ and Gln^6.55^ through H-bonds. Gln^6.55^ is substituted with a leucine in both ggTas2r1 and ggTas2r7, and is not conserved in the TAS2R family. Andrographolide is selective for ggTas2r7 and forms several H-bonds (i.e., with His^5.35^, Ser^5.38^, Ser^3.36^, and Gln^7.42^) and hydrophobic interactions with Met^3.32^ and Ile^5.42^. Position 7.42, a non-conserved residue both in chicken and human, is not found to be involved in ligand-interaction in ggTas2r1 (Di Pizio et al., 2017) and ggTas2r2. Interestingly, the interaction pattern of selective compounds is different from what we observed for the promiscuous ligands.

The predicted binding poses of promiscuous compounds are compatible in all three ggTas2rs, with position 6.51 involved in polar interaction and positions 3.32 and 5.42 in hydrophobic interactions. Interestingly, Trp/Phe^3.32^ is conserved in TAS2Rs, as well as a polar residue is present in position 6.51 and a hydrophobic residue in 5.42 (Table 1). Comparing the binding modes of promiscuous ligands into the three receptors we could underline interesting differences due to: (1) differences in the amino acid sequence, such as the one of position 5.38 - the presence of an aromatic residue in this position in both ggTas2r1 and ggTas2r2 (Phe and Tyr, respectively) allows for aromatic interactions with ligands within those receptors, which are not possible with Ser^5.58^ of ggTas2r7; (2) different residue arrangements, such as the case of Asn^3.36^ - this position is conserved between ggTas2r1 and ggTas2r2, but because of the different environment around the residue, Asn^3.36^ is involved in direct H-bond interactions with the agonists in ggTas2r1, whereas it is involved in intramolecular interactions with Tyr^6.47^ and/or Trp^3.32^ in ggTas2r2, and is not always available for direct ligand-binding. On the contrary, selective compounds tend to interact with non-conserved residues, allowing us to identify ggTas2r1-, ggTas2r2-, and ggTas2r7-specific residues responsible for selectivity: Lys^3.29^ and Asn^3.36^ as ggTas2r1-specific residues; Gln^6.55^ as ggTas2r2-specific residue; Ser^5.38^ and Gln^7.42^ as ggTas2r7-specific residues.

### ggTas2r selectivity of quinine analogs

**Figure 3:**
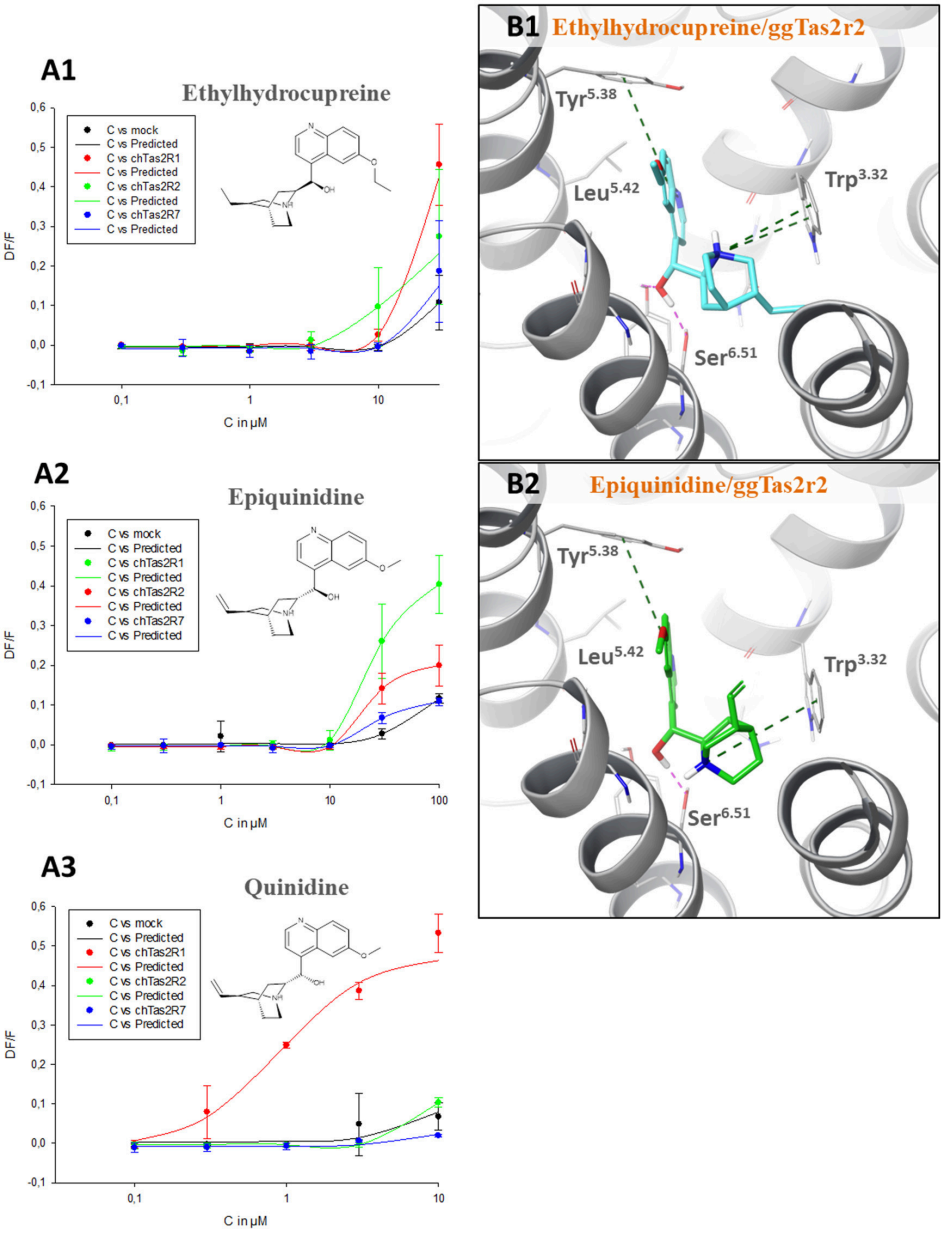
**(A)** Dose-response relations for ethylhydrocupreine **(A1)**, epiquinidine **(A2)**, and quinidine **(A3)** in cells expressing ggTas2r1, −2, and −7. The y-axis shows the relative fluorescence changes (ΔF/F), and the x axis the ligand concentration (C) in μM. Two experiments performed in duplicates were used for the calculations. The curves related to ggTas2r1 activation have been already published (Di Pizio et al., 2017). **(B)** Predicted binding modes of ethylhydrocupreine (in cyan, **B1**) and epiquinidine (in green, **B2**) into ggTas2r2 binding pocket. H-bond interactions are shown as magenta dotted lines, π-π interactions as green dashed lines.

Quinine analogs, i.e., quinidine, epiquinidine and ethylhydrocupreine, are an example of structurally similar chemicals that elicit different receptor activations. They have been recently found to activate ggTas2r1 with different threshold concentrations (~0.3 μM quinidine, ~10 μM epiquinidine and ethylhydrocupreine) (Di Pizio et al., 2017). To characterize their selectivity profile toward the entire ggTas2r family, we carried out calcium-mobilization assays using these compounds toward ggTas2r2 and ggTas2r7 as well (Figure 3A). All tested compounds are more selective toward ggTas2r1 than toward ggTas2r2 and ggTas2r7, as shown by activation patterns. Epiquinidine and ethylhydrocupreine can still activate ggTas2r2 and ggTas2r7, whereas quinidine is a selective ggTas2r1 agonist. As described previously, quinidine binds ggTas2r1 through an H-bond with Asn^3.36^ and hydrophobic interactions with Phe^3.32^, Phe^5.38^, Leu^5.42^, Tyr^6.47^ (Di Pizio et al., 2017). This binding mode is not permitted in ggTas2r2 (where Asn^3.36^ assumes a different conformation) and in ggTas2r7 (where Asn^3.36^ is replaced with a shorter Ser). Instead, in complex with ggTas2r1, epiquinidine and ethylhydrocupreine form H-bonds with Asn^6.51^, π-π stacking interaction with Phe^5.38^, and hydrophobic interactions with Leu^5.42^. Indeed, the predicted binding modes of these compounds in ggTas2r2 show a π-π stacking interaction with Tyr^5.38^ that is not allowed in the ggTas2r7 binding site (Figure 3B).

### Relations and overlaps between predicted bitter compounds for each ggTas2r

The ggTas2r models were next used to virtually screen molecules from BitterDB (Wiener et al., 2012) that are bitter for human, but have not yet been tested against ggTas2rs (642 compounds in total). To take the flexibility of the binding site residues into consideration, we used all agonist-bound models built for each ggTas2r subtype (the superimposition of all models is shown in Supplementary Figure S3). Pharmacophore modeling was employed as a filter prior to the docking screening. Pharmacophores are able to rationalize the receptor activity analyzing the ligand chemical structures (Martin et al., 2016), and pharmacophore-based virtual screening are generally very fast compared to high-throughput docking (Cheng et al., 2012). The pharmacophore filter allowed us to eliminate compounds that would have been obvious outliers and to submit to the docking analysis only compounds that have the molecular determinants responsible for receptor activity, speeding the screening process.

**Figure 4:**
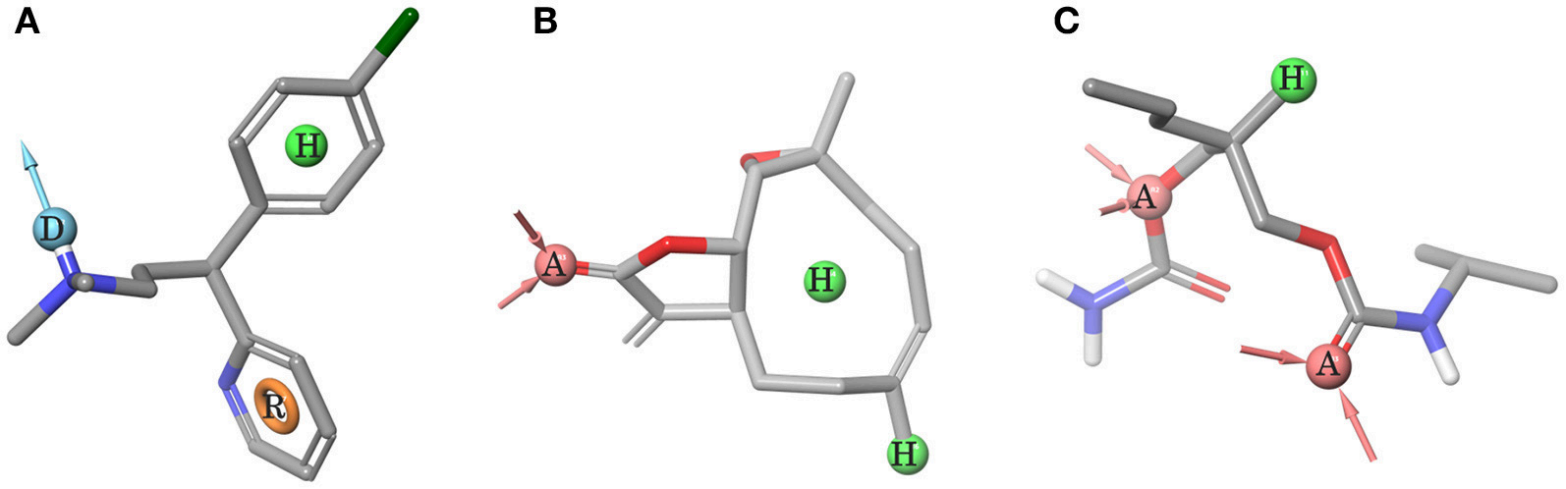
Pharmacophore models. **(A)** ggTas2r1 pharmacophore DHR (=H-Bond donor, hydrophobic feature, aromatic ring), reference structure chlorpheniramine, Sensitivity = 0.86, Specificity = 0.75. **(B)** ggTas2r2 pharmacophore AHH (=H-bond acceptor, hydrophobic feature, hydrophobic feature), reference structure quinine, Sensitivity = 0.67, Specificity = 0.62. **(C)** ggTas2r7 pharmacophore AAH (=H-bond acceptor, H-bond acceptor, hydrophobic feature), reference structure carisoprodol, Sensitivity = 0.78, Specificity = 0.68. Acceptor (red) and donor (cyan) features are shown as spheres and vectors, hydrophobic feature as green sphere, and aromatic feature as orange ring.

Pharmacophores built to predict ggTas2r1, ggTas2r2, and ggTas2r7 activity are represented in Figures 4A–C, respectively. ggTas2r1 pharmacophore, with chlorpheniramine as reference structure, is made of donor (D), hydrophobic (H) and aromatic (R) features, which correspond to the ligand groups that interact with Asn^3.36^, Phe^3.32^, and Phe^5.38^, respectively, in the chlorpheniramine/ggTas2r1 binding pose. Parthenolide is the reference compound of ggTas2r2 pharmacophore, where the acceptor (A) feature maps the carbonyl oxygen H-bonding with Asn^3.36^ and the hydrophobic (H) features match the aliphatic moieties of the ligands responsible for hydrophobic contacts with Trp88^3.32^ and Tyr180^5.38^. Two acceptors and one hydrophobic feature were found as determinants for ggTas2r7 activation. These features map the carisoprodol groups interacting with Ser98^3.36^, Gln275^7.42^, and Leu254^6.55^.

**Figure 5:**
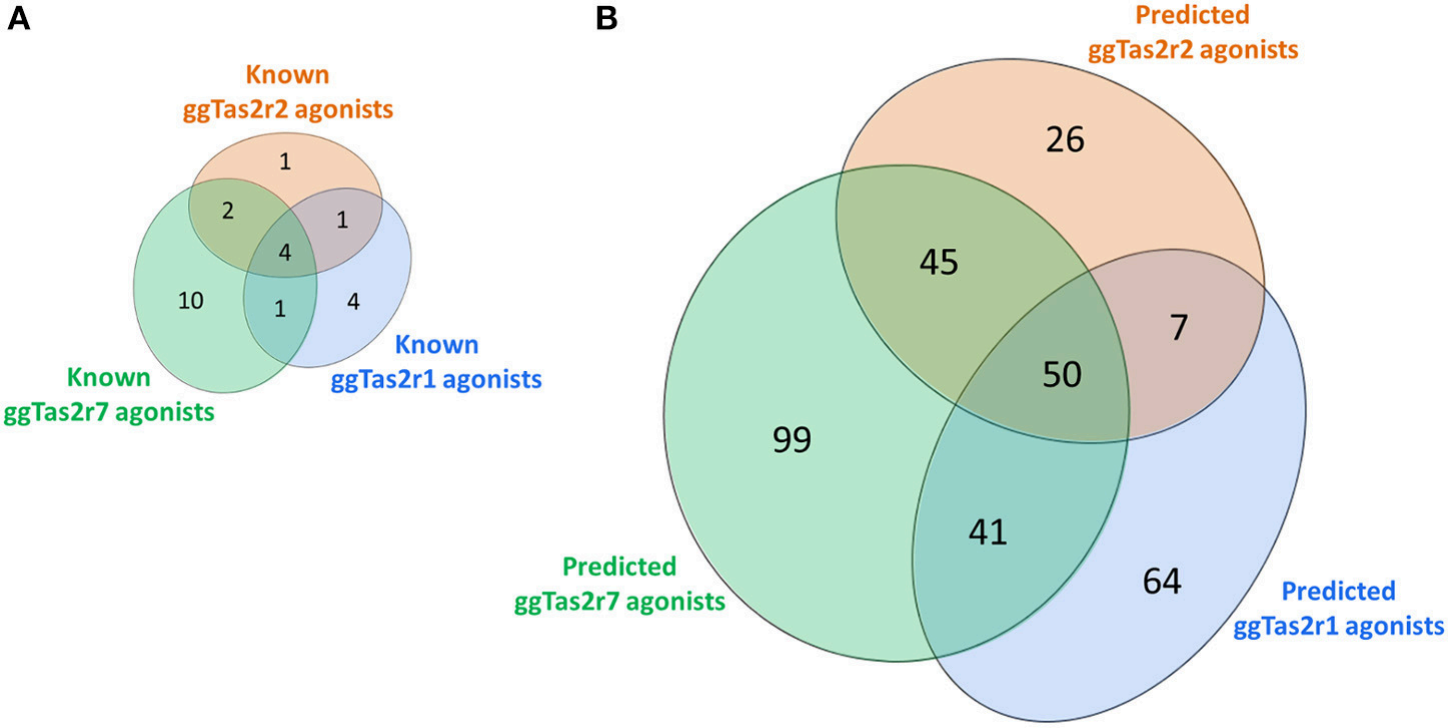
Venn diagrams of known **(A)** and predicted **(B)** ggTas2r agonists. The portions of ggTas2r1, −2, and −7 agonists are colored in blue, orange and green, respectively.

The screening of BitterDB against these pharmacophore models allowed for the selection of 165, 144, and 275 compounds as potential active compounds toward ggTas2r1,−2, and−7, respectively. Through docking, potential ggTas2r1 agonists were reduced to 162, equaling 25% of the database, the predicted ggTas2r2 agonists were reduced to 128, equaling 20% of screened library, and potential ggTas2r7 agonists were reduced to 236, equaling 37% of the screened compounds. In total, 332 of the 642 screened compounds are predicted to be bitter for chicken (52%). Interestingly, the percentage of the molecules selected with the virtual screening for each receptor is similar to that observed for the experimental screening, where ggTas2rs responded to 50% of tested molecules, ggTas2r1 and ggTas2r2 recognized ~20% of the compounds, and ggTas2r7 ~40% (Behrens et al., 2014). The relations between known and predicted compounds for each ggTas2r are represented in the Venn diagrams in Figure 5. Selected compounds, their pharmacophore and docking scores are reported in Supplementary Tables S1–S3.

## Conclusions

In this study, we analyzed the current data on bitter compounds for chicken to unravel the molecular mechanism of ligand recognition and selectivity in chicken bitter taste receptors. Detailed structural models of the ligand-binding pockets were obtained with homology modeling and induced-fit docking techniques and allowed for the identification of residue positions that may confer subtype selectivity. Integrating *in silico* and *in vitro* approaches we characterized the selectivity profiles of quinidine, epiquinidine and ethylhydrocupreine toward ggTas2rs. We next used ggTas2r models to screen BitterDB compounds and we predicted that ~50% of compounds known to be bitter for human may activate chicken receptors. Predicted ggTas2rs agonists could be valuable for *in vitro* and *in vivo* experiments, contributing to our understanding of bitter perception in chickens and, consequently, leading to the improvement of chicken feed. Moreover, we provide a methodology that can be applied to identify residue positions that may confer bitter taste receptor selectivity in humans.

## Methods

### Structure-based analysis

ggTas2r1, ggTas2r2, and ggTas2r7 sequences were aligned using the webserver MAFFT (Katoh and Standley, 2013). ggTas2r1 has 58% sequence similarity (identity 35%) with ggTas2r2 and 46% sequence similarity (identity 25%) with ggTas2r7; ggTas2r2 has 41% sequence similarity (identity 23%) with ggTas2r7. Homology modeling was performed with Prime (version 4.2, Schrödinger, LLC, New York, NY, 2015) (Jacobson et al., 2002, 2004) using as template ggTas2r1 previously modeled and refined in complex with quinine (Di Pizio et al., 2017). Protein structures were then optimized with the Protein Preparation Wizard tool from Schrödinger at physiological pH. Induced-fit Docking simulations were run to investigate the binding modes of quinine, diphenidol, chlorpheniramine, parthenolide, yohimbine, coumarin, caffeine, epiquinidine and ethylhydrocupreine with ggTas2r2; and of quinine, diphenidol, chlorpheniramine, parthenolide, yohimbine, chloramphenicol, amarogentin, absinthin, andrographolide, colchicine and carisoprodol with ggTas2r7. Glide (version 6.9) and Prime (version 4.2) were used for the Induced-Fit Docking protocol available in the Schrödinger software suite 2015-4 (Friesner et al., 2004; Zhu et al., 2007). The grid box was built at 10 Å from quinine in the quinine/ggTas2r1 complex. The van der Waals scaling of receptors and ligands were 0.5. Side chains of residues within 4.0 Å of the ligand were refined. The Extended Sampling protocol and OPLS 2005 force field were used. The docking was performed with the Standard Precision (SP) mode of Glide. Glide score, combined with visual inspection, was used to select the docking poses. The Ligand Interaction Diagram available in Maestro (version 10.4, Schrödinger, LLC, New York, NY, 2015) was used to create the 2D representations of predicted binding modes shown in Figure 2.

### Ligand-based analysis

Compounds and data activities reported in Table S2 of Behrens et al. (2014) were used to build the pharmacophore models using Phase (version 4.3, Schrödinger, LLC, New York, NY, 2015) (Dixon et al., 2006). Following features were modified: in donor (D) feature the setting [#1]([NH;X4]([#6;X4])([#6;X4])([#6;X4])) was set not to be excluded, the hydrophobic (H) feature was set to map both hydrophobic and aromatic moieties. The minimum intersite distance for features was set at 1 Å. 7 active (chloramphenicol, chloroquine, chlorpheniramine, diphenhydramin, diphenidol, nicotin, quinine) and 24 inactive molecules (absinthin, acetaminophen, alpha-thujone, amarogentin, andrographolide, brucine, caffeine, camphor, carisoprodol, colchicine, cycloheximide, denatonium, erythromycin, falcarindiol, ginkgolide A, limonin, noscapine, parthenolide, phenylthiocarbamide, quassin, saccharin, strychnine, thiamine, yohimbine) were used to build ggTas2r1 pharmacophore model DHR (one donor, one hydrophobic, and one aromatic feature). 6 actives (caffeine, chlorpheniramine, coumarin, diphenidol, parthenolide, quinine) and 26 inactives (absinthin, acetaminophen, alpha-thujone, amarogentin, andrographolide, azathioprine, brucine, camphor, carisoprodol, colchicine, cycloheximide, denatonium, diphenidramine, erythromycin, falcarindiol, ginkgolide A, limonin, nicotine, noscapine, phenylthiocarbamide, picrotoxinin, quassin, saccharin, strychnine, thiamine) were used to build the ggTas2r2 pharmacophore model AHH (one acceptor group and two hydrophobic features). 11 active (absinthin, amarogentin, andrographolide, carisoprodol, chloramphenicol, diphenidol, parthenolide, quinine, chlorpheniramine, colchicine, yohimbine) and 18 inactive molecules (acetaminophen, azathioprine, brucine, caffeine, camphor, chloroquine, coumarin, cycloheximide, denatonium, diphenidramine, falcarindiol, limonin, nicotine, noscapine, phenylthiocarbamide, saccharin, strychnine, thiamine) were used to build the ggTas2r7 pharmacophore model AAH (two acceptor and one hydrophobic features). Excluded volumes were added to each pharmacophore model around the reference structure (2 Å between the ligand and the shell). The sensitivity and specificity (SP) of the model were evaluated by the Equations (1) and (2):

(1) $$\begin{array}{r} Sensitivity=\frac{TP}{TP+FN} \end{array}$$

(2) $$\begin{array}{r} Specificity=\frac{TN}{TN+FP} \end{array}$$

where TP, TN, FP, and FN stand for the number of true positives, true negatives, false positives, and false negatives, respectively.

### Virtual screening

BitterDB compounds were prepared with LigPrep (version 3.6, Schrödinger, LLC, New York, NY, 2015) through the generation of stereoisomers and protonation states at pH 7 ± 0.5 and then with Phase (version 4.3, Schrödinger, LLC, New York, NY, 2015) to generate the 3D Phase Database, where for each molecule, pharmacophore sites and 100 conformers were calculated. The database was screened against ggTas2r1, ggTas2r2, and ggTas2r7 pharmacophore models, setting at least two features to be mapped and applying a fitness threshold of 0.4 (Dixon et al., 2006). Molecules filtered with the pharmacophore screening were passed to the docking analysis with the Virtual Screening Workflow, which allowed us to use more receptors simultaneously. We used both the models of ggTas2r1 in complex with agonists built in our previous work and the models of ggTas2r2 and ggTas2r7 in complex with agonists built here, as detailed above. Glide score threshold of 4.0 kcal/mol was applied to select docking results.

### Calcium-mobilization assays

ggTas2r1, ggTas2r2, and ggTas2r7 constructs, as well as empty expression vectors as controls were transiently transfected in HEK 293T cells stably expressing the chimeric Gα protein Gα16gust44, which were grown in 96-well plates exactly as described previously (Behrens et al., 2014). About 24 h after transfection, the cells were loaded with the calcium-sensitive dye Fluo4-AM in the presence of 2.5 mM probenecid, washed and placed in a fluorometric imaging plate reader (FLIPR-tetra, Molecular devices). Different concentrations of the bitter compounds (Sigma-Aldrich, Taufkirchen) were automatically applied and changes in fluorescence were monitored. A second application of 100 nM SST-14 stimulating endogenous somatostatin receptors was included as vitality control. Dose-response relations were calculated with SigmaPlot.

## Author contributions

ADP and MYN conceived the research. ADP and NS performed the ligand-based analysis; ADP conducted the structure-based experiments and wrote the manuscript. MB performed the *in vitro* experiments. MB and WM designed and analyzed the *in vitro* experiments. All authors reviewed the manuscript.

### Conflict of interest statement

The authors declare that the research was conducted in the absence of any commercial or financial relationships that could be construed as a potential conflict of interest.
