# Supplementary material for: Molecular Features Underlying Selectivity in Chicken Bitter Taste Receptors
Source: Front Mol Biosci. 2018 Jan 31;5:6. doi: 10.3389/fmolb.2018.00006 (PMC5797744; doi:10.3389/fmolb.2018.00006)
Supplement: Supplementary file 1 [file Table1.docx]

**Supplementary Information**

**Molecular features underlying selectivity in chicken bitter taste receptors**

Antonella Di Pizio, Nitzan Shy, Maik Behrens, Wolfgang Meyerhof and Masha Y. Niv

**Supplementary Figure S1: Matrix of residues involved in ligand-interaction.** Boxes are colored when the residue is involved in interaction. Blue color is used for ggTas2r1, orange for ggTas2r2, green for ggTas2r7. P=promiscuous ligands, I-P=ligands with intermediate promiscuity, S=selective ligands.


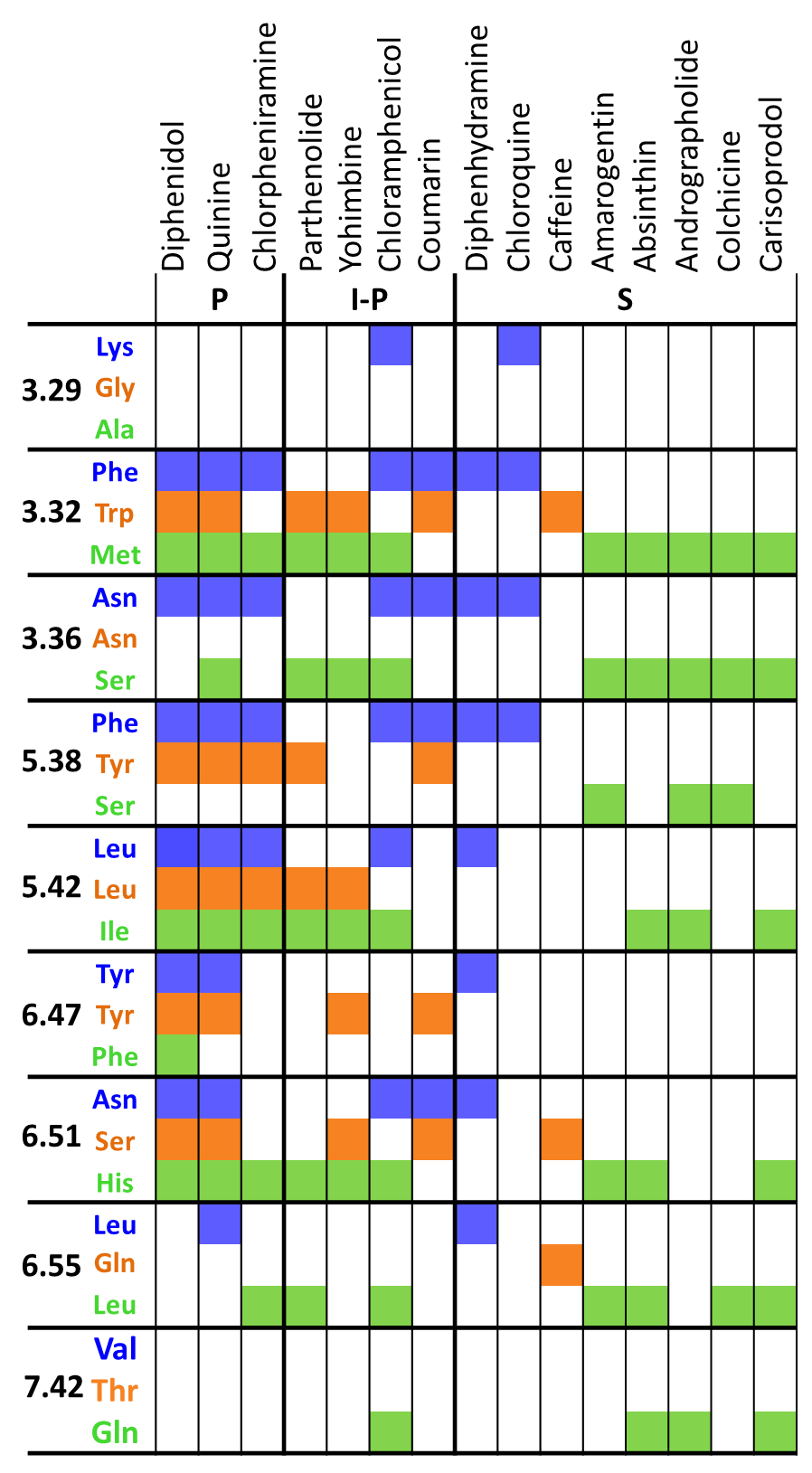


**Supplementary Figure S2:** 3D representation of predicted binding modes of promiscuous (panel A), intermediate-promiscuous (panel B) and selective compounds (panel C) into the ggTas2r binding sites. H-bond interactions are shown as magenta dashed lines.

**
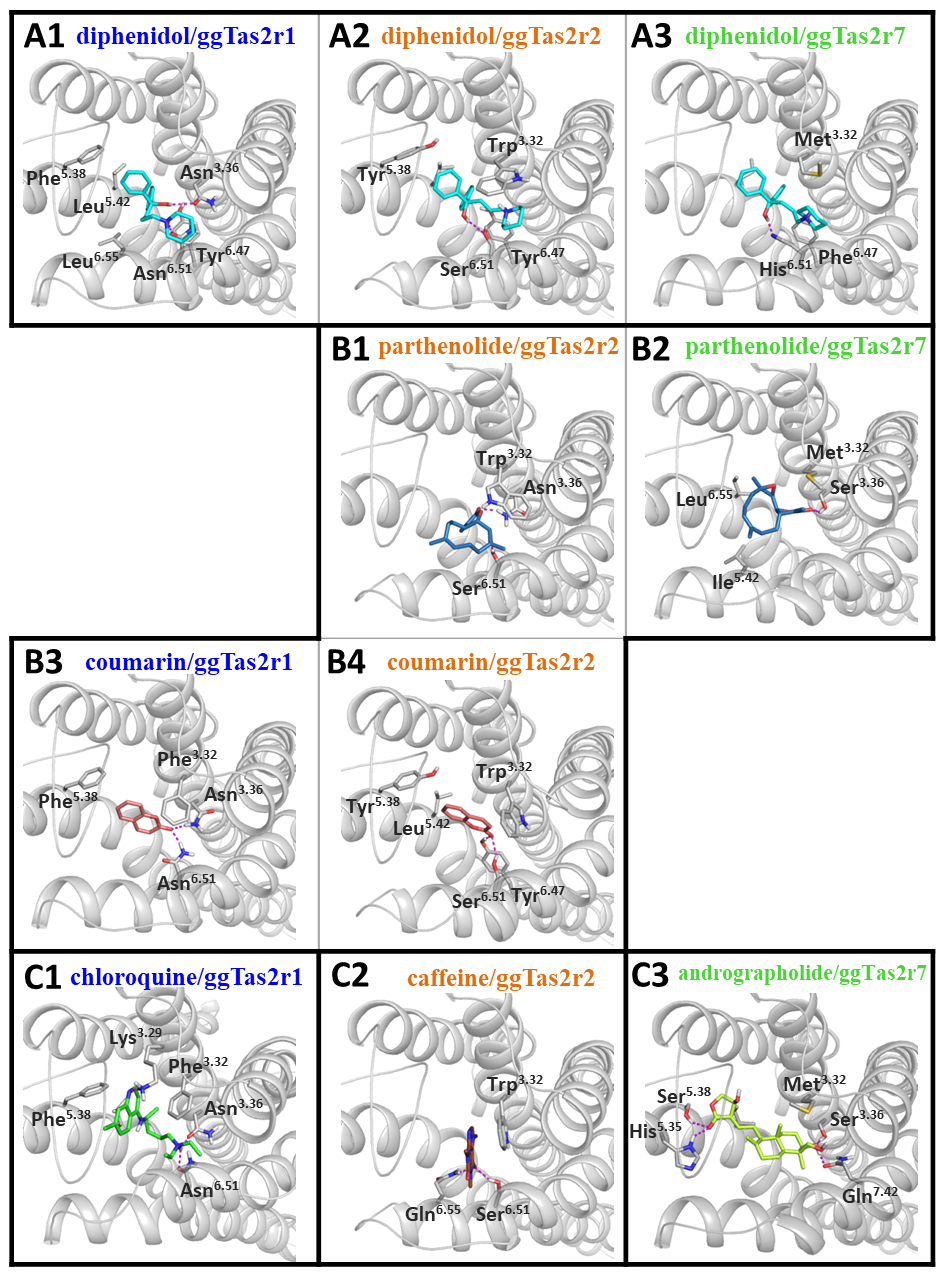
**

**Supplementary Figure S3:** **A)** **Superimposition of all analyzed complexes of ggTas2r1 with its agonists:** quinine (magenta), diphenidramine (yellow), diphenidol (cyan), chlorpheniramine (violet), chloramphenicol (orange), chloroquine (green), coumarin (red). Figure adapted from Di Pizio et. al Supplementary Figure S5 ([Di Pizio et al. 2017](#_ENREF_1)). **B)** **Superimposition of all analyzed complexes of ggTas2r2 with its agonists:** quinine (magenta), diphenidol (cyan), chlorpheniramine (violet), coumarin (red), caffeine (brown), parthenolide (blue), yohimbine (dark green). **C)** **Superimposition of all analyzed complexes of ggTas2r7 with its agonists:** absinthin (gray), chloramphenicol (orange), quinine (magenta), chloramphenicol (orange), diphenidol (cyan), chlorpheniramine (violet), coumarin (red), caffeine (brown), parthenolide (blue), yohimbine (dark green), carisoprodol (pink), amarogentin (light blue), andrographolide (green), colchicine (yellow).


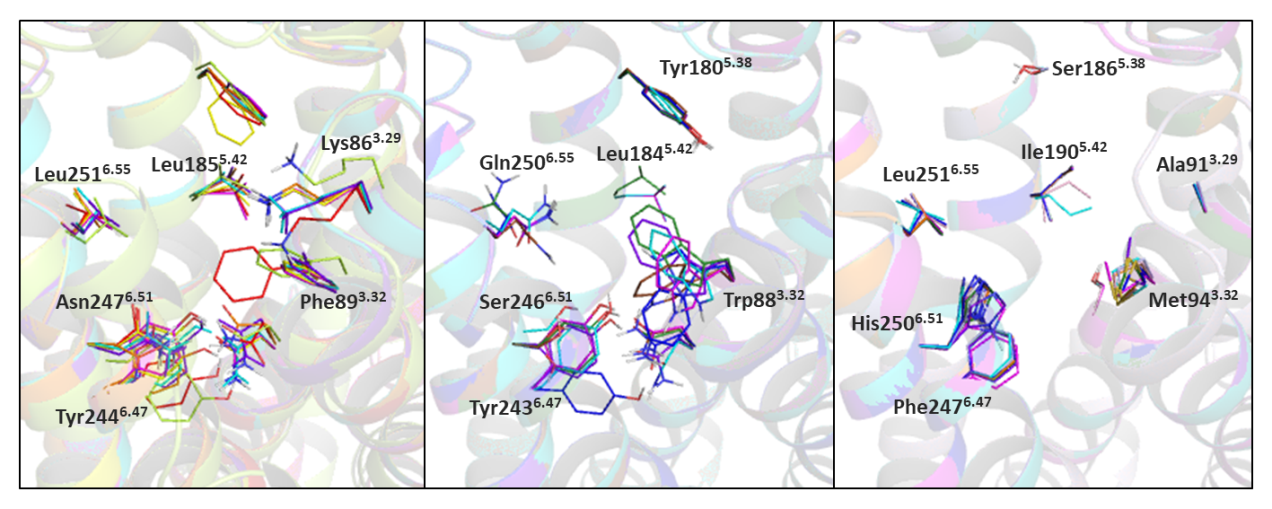


**Supporting Table S1:** BitterDB ID, fitness and Glide scores of compounds predicted to be active towards ggTas2r1

| BitterDB ID | Name | Fitness score | Glide score (kcal/mol) |
| --- | --- | --- | --- |
| 896 | Liquiritigenin | 0.413 | -11.051 |
| 829 | Phe-trp | 1.9 | -10.172 |
| 909 | Phloretinb | 0.422 | -9.502 |
| 134 | Cinnamedrine | 0.401 | -9.3 |
| 828 | Leu-trp | 0.422 | -9.05 |
| 65 | Flufenamic Acid | 0.419 | -8.9 |
| 88 | 8-prenylnaringenin | 1.987 | -8.835 |
| 371 | Pentobarbital Sodium salt | 0.422 | -8.789 |
| 560 | Cycrimine Hydrochloride | 2.562 | -8.77 |
| 232 | Harman | 0.405 | -8.731 |
| 672 | Umbelliferones | 0.412 | -8.726 |
| 892 | Eriodictyolb | 0.422 | -8.712 |
| 425 | Propallylonal | 0.423 | -8.45 |
| 587 | Hexethal Sodium | 0.421 | -8.447 |
| 402 | Picoperine Hydrochloride | 2.031 | -8.414 |
| 694 | Doxepin | 2.104 | -8.411 |
| 830 | Trp-leu | 0.411 | -8.397 |
| 937 | Mefenamic acid | 0.423 | -8.339 |
| 802 | (+/-) Equol | 0.421 | -8.334 |
| 615 | Butalbital | 0.422 | -8.31 |
| 443 | Quebrachamine | 0.411 | -8.255 |
| 120 | Propylthiouracil | 0.422 | -8.248 |
| 396 | Phenytoin | 2.091 | -8.236 |
| 715 | A,a-dimethylphenethyl alcohol | 0.41 | -8.224 |
| 893 | Flavanone | 0.422 | -8.213 |
| 881 | Datiscetinb | 0.413 | -8.097 |
| 813 | Epicatechin | 0.413 | -8.091 |
| 644 | Naringenin | 0.413 | -8.088 |
| 664 | 13-hydroxylupanine | 0.422 | -8.064 |
| 230 | Amobarbital Sodium salt | 0.422 | -8.031 |
| 695 | Thioacetanilide | 0.422 | -8.026 |
| 162 | Cyclobarbital | 1.836 | -7.989 |
| 385 | Phenobarbital | 1.836 | -7.989 |
| 411 | Piperidione | 0.422 | -7.959 |
| 580 | Chlorhexadol | 0.418 | -7.958 |
| 832 | Trp-trp | 1.241 | -7.957 |
| 234 | Heptabarbital | 0.419 | -7.946 |
| 577 | Butallylonal Sodium salt | 0.423 | -7.917 |
| 603 | Barbital Sodium salt | 0.418 | -7.911 |
| 103 | Arbutin | 0.423 | -7.889 |
| 413 | Pipradrol Hydrochloride | 2.005 | -7.881 |
| 653 | (-)-Riboflavin | 2.114 | -7.877 |
| 574 | Azacyclonol Hydrochloride | 2.517 | -7.826 |
| 536 | Tramadol Hydrochloride | 0.418 | -7.821 |
| 324 | Methyprylon | 0.422 | -7.802 |
| 827 | L-tryptophan | 0.423 | -7.786 |
| 646 | Promethazine | 2.128 | -7.772 |
| 620 | Allobarbital | 0.422 | -7.771 |
| 235 | Hexobarbital Sodium salt | 0.423 | -7.751 |
| 389 | Phentolamine | 2.662 | -7.74 |
| 895 | Homoeriodictyolc | 0.42 | -7.727 |
| 884 | Herbacetin | 0.409 | -7.706 |
| 597 | Phenylmethylbarbituric Acid | 0.408 | -7.694 |
| 353 | Antazoline Phosphate | 2.275 | -7.678 |
| 163 | Cyclobarbital | 0.418 | -7.677 |
| 660 | Meranzin | 0.421 | -7.65 |
| 570 | Melilotoside | 0.422 | -7.607 |
| 301 | Meperidine Hydrochloride | 1.429 | -7.601 |
| 464 | Salicylamide | 0.421 | -7.557 |
| 37 | Benzoin | 0.421 | -7.546 |
| 436 | Pseudoephedrine | 0.418 | -7.534 |
| 335 | Naphazoline Hydrochloride | 0.41 | -7.531 |
| 315 | Methoxsalen | 0.422 | -7.529 |
| 607 | Benzilic Acid | 0.422 | -7.529 |
| 68 | Thiamine | 0.422 | -7.504 |
| 520 | Thiamine Hydrochloride | 0.422 | -7.504 |
| 7 | Quinidine | 0.417 | -7.481 |
| 325 | Anethole Trithione | 0.413 | -7.468 |
| 612 | Bupropion Hydrochloride | 0.42 | -7.415 |
| 596 | Phenallymal | 1.898 | -7.41 |
| 705 | L-histidine | 0.423 | -7.403 |
| 484 | Sulfamerazine Monosodium salt | 0.417 | -7.392 |
| 197 | Dyphylline | 0.423 | -7.369 |
| 93 | Dapsone | 0.401 | -7.352 |
| 613 | Butabarbital Sodium salt | 0.422 | -7.342 |
| 752 | L-phenylalanine | 0.417 | -7.335 |
| 754 | 6-methylcoumarin | 0.42 | -7.331 |
| 700 | Spartein Sulfate Pentahydrate | 0.422 | -7.33 |
| 57 | Benzamide | 0.413 | -7.291 |
| 494 | Sulfisoxazole | 0.419 | -7.252 |
| 693 | Lupinine | 0.421 | -7.233 |
| 897 | Pinocembrin | 0.422 | -7.203 |
| 104 | Helicin | 0.405 | -7.187 |
| 627 | Carbinoxamine Maleate | 2.268 | -7.171 |
| 95 | 1,10-phenanthroline | 0.422 | -7.146 |
| 206 | Etilefrin Hydrochloride | 0.423 | -7.137 |
| 387 | Phenprobamate | 0.423 | -7.116 |
| 517 | Thiacetazone | 0.419 | -7.087 |
| 599 | Vinbarbital Sodium | 0.423 | -7.067 |
| 684 | Ethyl Benzoate | 0.409 | -7.039 |
| 543 | Azintamide | 0.422 | -7.027 |
| 185 | Dimemorfan Phosphate | 0.422 | -7.023 |
| 616 | Butethal | 0.422 | -7.022 |
| 624 | Carbimazole | 0.423 | -7.001 |
| 119 | Phenylthiocarbamide (PTC) | 0.421 | -7 |
| 583 | Cyclopentobarbital | 0.423 | -6.967 |
| 703 | Atropine Sulfate monohydrate | 0.411 | -6.966 |
| 237 | Homidium Bromide | 0.415 | -6.916 |
| 748 | Propyl gallate | 0.405 | -6.915 |
| 645 | Phenyl beta-D-glucopyranoside | 0.422 | -6.898 |
| 835 | Gly-phe | 0.422 | -6.873 |
| 61 | Diphenhydramine | 2.408 | -6.849 |
| 584 | Diethylbromoacetamide | 0.422 | -6.838 |
| 170 | Dextroamphetamine Sulfate | 0.418 | -6.828 |
| 942 | Diclofenac | 0.403 | -6.809 |
| 130 | Orphenadrine | 2.36 | -6.775 |
| 142 | Clobutinol | 0.422 | -6.769 |
| 426 | Aprobarbital Sodium salt | 0.42 | -6.756 |
| 427 | Aprobarbital | 0.42 | -6.756 |
| 750 | Phenethyl alcohol | 0.422 | -6.75 |
| 579 | Chlorbetamide | 1.939 | -6.733 |
| 755 | 2-methoxy-4-methylphenol | 0.401 | -6.687 |
| 743 | Cinnamyl alcohol | 0.422 | -6.673 |
| 488 | Sulfamethoxazole | 0.422 | -6.671 |
| 591 | Narcobarbital | 0.42 | -6.668 |
| 785 | A-terpinyl anthranilate | 0.417 | -6.655 |
| 231 | Guaifenesin | 0.423 | -6.625 |
| 795 | Propyl 2-furanacrylate | 0.423 | -6.61 |
| 503 | Talbutal | 0.422 | -6.61 |
| 467 | Secobarbital Sodium | 0.422 | -6.603 |
| 716 | Ethyl phenylacetate | 0.423 | -6.588 |
| 461 | Salicin | 0.422 | -6.577 |
| 853 | 3‐isopropoxy‐1λ⁶,2‐benzothiazole‐1,1‐dione | 0.423 | -6.544 |
| 415 | Potassium Guaiacolsulfonate | 0.417 | -6.542 |
| 593 | Nealbarbital | 0.423 | -6.514 |
| 400 | Diethyl phthalate | 0.412 | -6.507 |
| 639 | Chloroprocaine | 0.42 | -6.495 |
| 344 | Nikethamide | 0.4 | -6.438 |
| 285 | Amylocaine Hydrochloride | 2.084 | -6.423 |
| 392 | Phenylephrine | 0.423 | -6.417 |
| 149 | Alverine | 1.46 | -6.372 |
| 564 | Ethylbenzhydramine Hydrochloride | 2.144 | -6.369 |
| 696 | Goitrin | 0.42 | -6.32 |
| 710 | Isoamyl salicylate | 0.418 | -6.314 |
| 304 | Meprobamate | 0.423 | -6.306 |
| 282 | Linamarin | 0.421 | -6.304 |
| 650 | Nicotine | 0.415 | -6.278 |
| 317 | Methoxyphenamine Hydrochloride | 0.42 | -6.269 |
| 533 | Tolazoline Hydrochloride | 0.423 | -6.264 |
| 200 | Epirizole | 0.408 | -6.252 |
| 546 | Valpromide | 0.423 | -6.239 |
| 209 | Fenproporex Hydrochloride | 0.416 | -6.216 |
| 313 | Methamphetamine Hydrochloride | 0.419 | -6.161 |
| 712 | Ethyl benzoylacetate | 0.416 | -6.131 |
| 709 | Isobutyl salicylate | 0.417 | -6.122 |
| 537 | Trapidil | 0.423 | -6.104 |
| 420 | Procaine Dihydrate | 0.422 | -6.069 |
| 161 | Cyclexedrine Hydrochloride | 0.423 | -5.964 |
| 259 | Acecarbromal | 0.409 | -5.841 |
| 834 | Gly-leu | 0.421 | -5.831 |
| 165 | Cyclopentamine Hydrochloride | 0.422 | -5.781 |
| 112 | N-ethylthiourea | 0.412 | -5.688 |
| 266 | Iodinated Glycerol | 0.422 | -5.64 |
| 751 | L-leucine | 0.412 | -5.62 |
| 303 | Mephenesin | 0.423 | -5.569 |
| 730 | 3-heptanol | 0.422 | -5.396 |
| 269 | L-Isoleucine | 0.416 | -5.117 |
| 718 | 3,7-dimethyl-1-octanol | 0.423 | -4.997 |
| 791 | Nerol | 0.422 | -4.735 |
| 725 | D-citronellol | 0.421 | -4.702 |
| 729 | 2-heptanol | 0.423 | -4.589 |
| 521 | Thiosinamine | 0.423 | -4.411 |
| 351 | D-Norleucine | 0.423 | -4.307 |

**Supporting Table S2:** BitterDB ID, fitness and Glide scores of compounds predicted to be active towards ggTas2r2

| BitterDB ID | Name | Fitness score | Glide score (kcal/mol) |
| --- | --- | --- | --- |
| 829 | Phe-trp | 1.19 | -9.411 |
| 321 | Methylergonovine Maleate | 1.462 | -9.273 |
| 653 | (-)-Riboflavin | 2.066 | -8.849 |
| 832 | Trp-trp | 1.228 | -8.832 |
| 523 | Tiaramide Hydrochloride | 2.049 | -8.473 |
| 820 | Glucobrassicin | 1.667 | -8.432 |
| 88 | 8-prenylnaringenin | 1.892 | -8.414 |
| 553 | Carnosol | 2.012 | -8.329 |
| 894 | Hesperetin | 2.115 | -8.237 |
| 680 | Omeprazole | 1.7 | -8.158 |
| 544 | Trimethoprim | 2.069 | -7.996 |
| 134 | Cinnamedrine | 1.728 | -7.985 |
| 582 | Bakankosin | 1.973 | -7.978 |
| 227 | Glucovanillin | 1.792 | -7.925 |
| 162 | Cyclobarbital | 1.439 | -7.91 |
| 385 | Phenobarbital | 1.439 | -7.91 |
| 131 | Tatridin B | 1.882 | -7.904 |
| 749 | Theobromine | 1.912 | -7.892 |
| 241 | Amodiaquin Dihydrochloride dihydrate | 1.722 | -7.833 |
| 586 | Garryine | 1.952 | -7.827 |
| 468 | Senecionine | 1.81 | -7.821 |
| 458 | Rhododendrin | 2.045 | -7.816 |
| 98 | Cycloheximide ; cycloheximid | 1.726 | -7.792 |
| 830 | Trp-leu | 1.494 | -7.778 |
| 151 | Codamine | 1.28 | -7.765 |
| 76 | Xanthohumol | 1.951 | -7.734 |
| 498 | Suxibuzone | 1.179 | -7.698 |
| 83 | cis-isohumulone | 2.17 | -7.664 |
| 11 | Humulon | 1.377 | -7.639 |
| 68 | Thiamine | 1.925 | -7.589 |
| 520 | Vitamin b1 ; Thiamine Hydrochloride | 1.925 | -7.589 |
| 664 | 13-hydroxylupanine | 1.779 | -7.583 |
| 934 | Pantoprazole | 1.853 | -7.573 |
| 66 | Haloperidol | 1.676 | -7.558 |
| 885 | Isorhamnetin | 1.822 | -7.549 |
| 895 | Homoeriodictyolc | 1.569 | -7.513 |
| 144 | Clonixin | 1.815 | -7.504 |
| 128 | Crispolide | 2.167 | -7.494 |
| 828 | Leu-trp | 1.196 | -7.468 |
| 74 | Colupulone | 1.319 | -7.45 |
| 163 | Cyclobarbital | 1.468 | -7.41 |
| 73 | Cohumulone | 1.86 | -7.403 |
| 45 | Artemorin | 1.965 | -7.381 |
| 48 | Cascarillin | 1.624 | -7.38 |
| 579 | Chlorbetamide | 2.27 | -7.371 |
| 603 | Barbital Sodium salt | 1.716 | -7.32 |
| 53 | Papaverine | 1.277 | -7.305 |
| 373 | Pentoxifylline | 1.859 | -7.303 |
| 583 | Cyclopentobarbital | 1.565 | -7.29 |
| 72 | Adhumulone | 1.72 | -7.265 |
| 405 | Picrocrocin, saffron-bitter | 2.108 | -7.265 |
| 234 | Heptabarbital | 1.676 | -7.259 |
| 200 | Epirizole | 1.772 | -7.2 |
| 42 | Arborescin | 2.131 | -7.191 |
| 852 | N,N-diethyl-6-nitro-1,1-dioxo-1,2-benzothiazol-3-amine | 1.662 | -7.165 |
| 106 | Sinigrin | 2.31 | -7.134 |
| 414 | Plaunotol | 1.588 | -7.094 |
| 43 | Arglabin | 2.005 | -7.085 |
| 615 | Butalbital | 0.923 | -7.058 |
| 785 | A-terpinyl anthranilate | 1.466 | -7.051 |
| 616 | Butethal | 1.755 | -7.05 |
| 85 | trans-isoadhumulone | 1.356 | -7.033 |
| 530 | Tinoridine Hydrochloride | 1.386 | -7.002 |
| 547 | Veatchine | 1.38 | -6.977 |
| 283 | Lincomycin Hydrochloride monohydrate | 1.978 | -6.903 |
| 315 | Methoxsalen | 1.98 | -6.878 |
| 596 | Phenallymal | 1.401 | -6.854 |
| 79 | trans-isohumulone | 1.15 | -6.828 |
| 577 | Butallylonal Sodium salt | 1.645 | -6.825 |
| 938 | Pemirolast | 1.912 | -6.812 |
| 845 | 6‐nitro‐2‐[(trichloromethyl)sulfanyl]‐2,3‐dihydro,2‐benzothiazole‐1,1,3‐trione | 1.358 | -6.81 |
| 486 | Sulfameter | 2.064 | -6.792 |
| 81 | Adlupulone | 1.229 | -6.785 |
| 230 | Amobarbital Sodium salt | 2.068 | -6.784 |
| 660 | Meranzin | 2.113 | -6.776 |
| 425 | Propallylonal | 2.03 | -6.762 |
| 537 | Trapidil | 1.621 | -6.76 |
| 613 | Butabarbital Sodium salt | 1.701 | -6.756 |
| 371 | Pentobarbital Sodium salt | 2.042 | -6.754 |
| 599 | Vinbarbital Sodium | 2.216 | -6.714 |
| 754 | 6-methylcoumarin | 1.862 | -6.706 |
| 593 | Nealbarbital | 2.008 | -6.705 |
| 587 | Hexethal Sodium | 2.351 | -6.666 |
| 491 | Sulfamethoxypyridazine | 2.003 | -6.653 |
| 298 | Meconin | 1.702 | -6.639 |
| 304 | Meprobamate | 1.651 | -6.627 |
| 503 | Talbutal | 1.689 | -6.563 |
| 467 | Secobarbital Sodium | 2.029 | -6.54 |
| 282 | Linamarin | 1.924 | -6.534 |
| 591 | Narcobarbital | 2.025 | -6.532 |
| 52 | Noscapine | 1.326 | -6.499 |
| 344 | Nikethamide | 1.842 | -6.49 |
| 426 | Aprobarbital Sodium salt | 1.742 | -6.48 |
| 427 | Aprobarbital | 1.742 | -6.48 |
| 494 | Sulfisoxazole | 1.871 | -6.47 |
| 539 | Tributyrin | 1.592 | -6.4 |
| 142 | Clobutinol | 1.685 | -6.372 |
| 543 | Azintamide | 2.011 | -6.366 |
| 620 | Allobarbital | 1.654 | -6.341 |
| 82 | cis-isocohumulone | 1.662 | -6.332 |
| 484 | Sulfamerazine Monosodium salt | 2.076 | -6.317 |
| 759 | 3-phenylpropyl isobutyrate | 1.267 | -6.275 |
| 855 | 2‐[2‐(dibutylamino)ethyl]‐2H‐1λ⁶,3λ⁶,2‐benzodithiazole-1,1,3,3‐tetrone | 1.637 | -6.275 |
| 387 | Phenprobamate | 1.291 | -6.174 |
| 580 | Chlorhexadol | 2.132 | -6.16 |
| 839 | N‐[(4‐isopropylphenyl)methylidene]hydroxylamine | 1.839 | -6.139 |
| 779 | 3-phenylpropyl acetate | 1.295 | -6.103 |
| 589 | Lactophenetide | 1.829 | -6.042 |
| 799 | Santalyl acetate | 2.263 | -6.03 |
| 259 | Acecarbromal | 1.838 | -5.984 |
| 823 | Alitame-ll isomer | 2.18 | -5.972 |
| 786 | Terpinyl propionate | 1.811 | -5.944 |
| 831 | Trp-phe | 1.412 | -5.932 |
| 790 | Terpinyl isovalerate | 1.653 | -5.855 |
| 777 | Terpinyl formate | 2.298 | -5.729 |
| 710 | Isoamyl salicylate | 1.996 | -5.615 |
| 266 | Iodinated Glycerol | 1.986 | -5.256 |
| 741 | Geranyl acetate | 2.036 | -5.246 |
| 760 | Phenethyl formate | 1.668 | -5.21 |
| 231 | Guaifenesin | 1.917 | -5.187 |
| 303 | Mephenesin | 1.887 | -5.128 |
| 744 | Cinnamyl formate | 1.824 | -4.998 |
| 742 | Geranyl formate | 2.152 | -4.784 |
| 791 | Nerol | 2.145 | -4.746 |
| 782 | Linalyl formate | 2.417 | -4.41 |
| 617 | Butethamine meta-Isomer hydrochloride | 1.74 | -4.408 |
| 773 | 6-methyl-5-hepten-2-one | 1.888 | -4.362 |
| 725 | D-citronellol | 2.164 | -4.123 |

**Supporting Table S3:** BitterDB ID, fitness and Glide scores of compounds predicted to be active towards ggTas2r7

| BitterDB ID | Name | Fitness score | Glide score (kcal/mol) |
| --- | --- | --- | --- |
| 75 | Isoxanthohumol | 1.537 | -8.346 |
| 134 | Cinnamedrine | 0.42 | -8.303 |
| 800 | Genistein | 0.422 | -8.118 |
| 828 | Leu-trp | 0.402 | -8.055 |
| 676 | Pirenzapin | 0.406 | -7.991 |
| 909 | Phloretinb | 0.422 | -7.889 |
| 814 | Epigallocatechin | 0.422 | -7.805 |
| 88 | 8-prenylnaringenin | 1.453 | -7.73 |
| 809 | Catechin | 0.423 | -7.701 |
| 74 | Colupulone | 0.41 | -7.676 |
| 84 | cis-isoloadhumulone | 0.411 | -7.667 |
| 824 | Coumestrol | 0.417 | -7.652 |
| 644 | Naringenin | 0.422 | -7.634 |
| 896 | Liquiritigenin | 0.42 | -7.592 |
| 898 | Fustin | 0.41 | -7.583 |
| 499 | Swertiamarin | 1.747 | -7.56 |
| 900 | (+)-taxifolin | 0.423 | -7.537 |
| 813 | Epicatechin | 0.422 | -7.513 |
| 11 | Humulon | 1.995 | -7.443 |
| 897 | Pinocembrin | 0.422 | -7.434 |
| 892 | Eriodictyolb | 0.423 | -7.433 |
| 127 | Cnicin | 2.024 | -7.405 |
| 653 | (-)-Riboflavin | 0.423 | -7.4 |
| 396 | Phenytoin | 0.41 | -7.364 |
| 85 | trans-isoadhumulone | 1.626 | -7.36 |
| 82 | cis-isocohumulone | 0.409 | -7.347 |
| 680 | Omeprazole | 0.41 | -7.346 |
| 664 | 13-hydroxylupanine | 0.422 | -7.335 |
| 749 | Theobromine | 1.755 | -7.32 |
| 191 | Aminophylline | 0.416 | -7.314 |
| 516 | Theophylline Sodium acetate | 0.416 | -7.314 |
| 697 | Enalapril | 0.419 | -7.312 |
| 197 | Dyphylline | 0.423 | -7.292 |
| 94 | denatonium benzoate | 0.422 | -7.253 |
| 283 | Lincomycin Hydrochloride monohydrate | 1.437 | -7.253 |
| 657 | Cynaropicrin | 1.788 | -7.237 |
| 339 | Neoquassin | 1.873 | -7.225 |
| 334 | Moveltipril, calcium salt | 0.4 | -7.17 |
| 894 | Hesperetin | 0.422 | -7.147 |
| 682 | Prednisone | 1.749 | -7.122 |
| 895 | Homoeriodictyolc | 1.692 | -7.122 |
| 73 | Cohumulone | 2.088 | -7.105 |
| 128 | Crispolide | 0.422 | -7.081 |
| 557 | Amprotropine Phosphate | 1.512 | -7.08 |
| 458 | Rhododendrin | 1.904 | -7.057 |
| 547 | Veatchine | 0.422 | -6.985 |
| 48 | Cascarillin | 1.925 | -6.963 |
| 553 | Carnosol | 2.334 | -6.952 |
| 413 | Pipradrol Hydrochloride | 0.421 | -6.937 |
| 551 | Warfarin Sodium salt | 0.422 | -6.928 |
| 692 | Cinchonine | 0.423 | -6.925 |
| 548 | Verbenalin | 0.419 | -6.881 |
| 233 | Helenalin | 1.986 | -6.846 |
| 579 | Chlorbetamide | 1.413 | -6.816 |
| 544 | Trimethoprim | 0.421 | -6.811 |
| 582 | Bakankosin | 1.987 | -6.806 |
| 688 | Clindamycin | 1.948 | -6.789 |
| 373 | Pentoxifylline | 1.869 | -6.788 |
| 645 | Phenyl beta-D-glucopyranoside | 0.423 | -6.769 |
| 785 | A-terpinyl anthranilate | 0.421 | -6.751 |
| 933 | Herbolide D | 1.993 | -6.748 |
| 7 | Quinidine | 0.412 | -6.731 |
| 405 | Picrocrocin | 2.08 | -6.728 |
| 235 | Hexobarbital Sodium salt | 2.017 | -6.72 |
| 411 | Piperidione | 2.076 | -6.72 |
| 796 | 3-methyl-2-phenylbutyraldehyde | 0.422 | -6.71 |
| 98 | Cycloheximide | 1.46 | -6.693 |
| 200 | Epirizole | 1.689 | -6.638 |
| 603 | Barbital Sodium salt | 2.017 | -6.632 |
| 163 | Cyclobarbital | 2.023 | -6.618 |
| 80 | trans-isocohumulone | 1.438 | -6.614 |
| 357 | Oxeladin | 2.048 | -6.608 |
| 227 | Glucovanillin | 1.464 | -6.606 |
| 461 | Salicin | 0.423 | -6.594 |
| 767 | O-methoxybenzaldehyde | 0.414 | -6.587 |
| 674 | Ofloxacin | 0.422 | -6.585 |
| 151 | Codamine | 1.908 | -6.584 |
| 144 | Clonixin | 0.423 | -6.579 |
| 596 | Phenallymal | 2.218 | -6.572 |
| 104 | Helicin | 0.422 | -6.569 |
| 819 | Progoitrin | 2.194 | -6.566 |
| 938 | Pemirolast | 0.422 | -6.553 |
| 72 | Adhumulone | 2.012 | -6.551 |
| 484 | Sulfamerazine Monosodium salt | 1.857 | -6.543 |
| 549 | Viquidil Hydrochloride | 1.625 | -6.526 |
| 162 | Cyclobarbital | 1.878 | -6.52 |
| 385 | Phenobarbital | 1.878 | -6.52 |
| 805 | Alpha-tetralone | 0.421 | -6.504 |
| 634 | Chenodiol | 0.422 | -6.499 |
| 703 | Atropine Sulfate monohydrate | 0.42 | -6.498 |
| 45 | Artemorin | 0.423 | -6.488 |
| 120 | Propylthiouracil | 0.423 | -6.484 |
| 852 | 3‐(diethylamino)‐6‐nitro‐1λ⁶,2‐benzothiazole‐1,1‐dione | 0.422 | -6.482 |
| 494 | Sulfisoxazole | 2.06 | -6.478 |
| 148 | Cocaine Hydrochloride | 0.41 | -6.462 |
| 216 | Fraxin | 1.514 | -6.434 |
| 125 | saccharin | 0.405 | -6.425 |
| 392 | Phenylephrine | 0.423 | -6.402 |
| 486 | Sulfameter | 1.516 | -6.4 |
| 468 | Senecionine | 1.953 | -6.387 |
| 324 | Methyprylon | 2.071 | -6.38 |
| 853 | 3‐isopropoxy‐1λ⁶,2‐benzothiazole‐1,1‐dione | 0.422 | -6.373 |
| 103 | Arbutin | 0.422 | -6.37 |
| 537 | Trapidil | 1.865 | -6.367 |
| 693 | Lupinine | 0.421 | -6.367 |
| 234 | Heptabarbital | 2.299 | -6.366 |
| 848 | 2-[3-(diethylamino)propyl]-1,1-dioxo-1,2-benzothiazol-3-one | 0.418 | -6.366 |
| 206 | Etilefrin Hydrochloride | 0.423 | -6.361 |
| 755 | 2-methoxy-4-methylphenol | 0.421 | -6.355 |
| 106 | Sinigrin | 2.199 | -6.351 |
| 158 | Coriamyrtin | 2.11 | -6.342 |
| 613 | Butabarbital Sodium salt | 2.131 | -6.342 |
| 43 | Arglabin | 1.918 | -6.34 |
| 465 | Santonin | 0.412 | -6.335 |
| 787 | A-methylbenzyl acetate | 0.42 | -6.331 |
| 407 | Picrotoxin | 1.917 | -6.327 |
| 597 | Phenylmethylbarbituric Acid | 0.422 | -6.326 |
| 299 | Menthone | 0.423 | -6.324 |
| 733 | Fenchyl alcohol | 0.412 | -6.324 |
| 387 | Phenprobamate | 0.416 | -6.303 |
| 116 | 6-Methyl-2-thiouracil | 0.421 | -6.293 |
| 570 | Melilotoside | 0.421 | -6.276 |
| 221 | Gentiobiose | 0.422 | -6.274 |
| 738 | D-camphor | 0.422 | -6.269 |
| 541 | Trimethadione | 0.409 | -6.268 |
| 583 | Cyclopentobarbital | 2.328 | -6.256 |
| 278 | Khellin | 1.353 | -6.232 |
| 668 | Gentianine | 1.914 | -6.23 |
| 599 | Vinbarbital Sodium | 2.181 | -6.228 |
| 491 | Sulfamethoxypyridazine | 0.415 | -6.227 |
| 290 | Lycopodine | 0.417 | -6.214 |
| 142 | Clobutinol | 0.42 | -6.186 |
| 712 | Ethyl benzoylacetate | 1.898 | -6.166 |
| 849 | 6‐nitro‐2‐(prop‐2‐en‐1‐yl)‐1λ⁶,2‐benzothiazole‐1,1,3‐trione | 0.418 | -6.166 |
| 410 | Pipemidic Acid | 1.443 | -6.149 |
| 230 | Amobarbital Sodium salt | 2.162 | -6.128 |
| 588 | Homocamfin | 0.423 | -6.128 |
| 425 | Propallylonal | 2.301 | -6.123 |
| 772 | Piperonyl acetate | 0.422 | -6.123 |
| 323 | Methylthiouracil | 0.421 | -6.121 |
| 687 | Cyclooctanone | 0.423 | -6.115 |
| 851 | 2-[2-(dibutylamino)ethyl]-1,1-dioxo-1,2-benzothiazol-3-one | 1.706 | -6.111 |
| 130 | orphenadrine | 0.411 | -6.107 |
| 847 | 2-[2-(diethylamino)ethyl]-1,1-dioxo-1,2-benzothiazol-3-one | 0.418 | -6.105 |
| 841 | 7- nitrosaccharin | 0.405 | -6.092 |
| 505 | Terpin hydrate | 0.422 | -6.078 |
| 577 | Butallylonal Sodium salt | 2.301 | -6.071 |
| 799 | Santalyl acetate | 0.41 | -6.059 |
| 344 | Nikethamide | 2.001 | -6.046 |
| 371 | Pentobarbital Sodium salt | 2.282 | -6.043 |
| 315 | Methoxsalen | 0.422 | -6.028 |
| 616 | Butethal | 2.286 | -6.025 |
| 332 | Morphine Hydrochloride | 0.415 | -6.022 |
| 503 | Talbutal | 2.335 | -6.02 |
| 768 | Phenethyl isovalerate | 0.421 | -6.017 |
| 298 | Meconin | 1.547 | -6.005 |
| 591 | Narcobarbital | 2.342 | -6.003 |
| 584 | Diethylbromoacetamide | 0.423 | -5.984 |
| 854 | 2‐[3‐(diethylamino)propyl]‐1λ⁶,3λ⁶,2‐benzodithiazole‐1,1,3,3‐tetrone | 0.423 | -5.972 |
| 543 | Azintamide | 1.712 | -5.956 |
| 735 | Ethyl maltol | 1.962 | -5.953 |
| 166 | Deferiprone | 0.415 | -5.925 |
| 224 | Ginkgolide A | 0.401 | -5.922 |
| 658 | Gallic aci | 0.421 | -5.917 |
| 587 | Hexethal Sodium | 2.243 | -5.913 |
| 122 | Acesulfame K | 1.75 | -5.911 |
| 536 | Tramadol Hydrochloride | 1.823 | -5.907 |
| 585 | Dioxypyramidon | 0.423 | -5.9 |
| 615 | Butalbital | 2.231 | -5.899 |
| 109 | Caprolactam | 0.423 | -5.878 |
| 765 | Piperonal | 0.413 | -5.865 |
| 759 | 3-phenylpropyl isobutyrate | 0.4 | -5.863 |
| 593 | Nealbarbital | 2.241 | -5.846 |
| 42 | Arborescin | 1.878 | -5.79 |
| 426 | Aprobarbital Sodium salt | 2.299 | -5.788 |
| 427 | Aprobarbital | 2.299 | -5.788 |
| 89 | Picrotoxinin | 1.877 | -5.784 |
| 488 | Sulfamethoxazole | 1.968 | -5.77 |
| 778 | Menthyl isovalerate | 0.423 | -5.765 |
| 578 | Camphotamide | 0.423 | -5.762 |
| 374 | Pentylenetetrazole | 0.422 | -5.76 |
| 758 | Phenethyl isobutyrate | 0.423 | -5.756 |
| 534 | Topiramate | 1.941 | -5.722 |
| 196 | Drotebanol | 0.423 | -5.701 |
| 659 | Laevuflex | 0.423 | -5.662 |
| 660 | Meranzin | 2.03 | -5.657 |
| 259 | Acecarbromal | 1.989 | -5.636 |
| 732 | D-fenchone | 0.419 | -5.63 |
| 737 | Glucose pentaacetate | 0.422 | -5.617 |
| 716 | Ethyl phenylacetate | 0.423 | -5.608 |
| 282 | Linamarin | 2.207 | -5.603 |
| 400 | Diethyl phthalate | 1.786 | -5.602 |
| 748 | Propyl gallate | 0.422 | -5.576 |
| 467 | Secobarbital Sodium | 2.247 | -5.575 |
| 317 | Methoxyphenamine Hydrochloride | 0.422 | -5.562 |
| 639 | Chloroprocaine | 0.422 | -5.554 |
| 304 | Meprobamate | 2.517 | -5.548 |
| 709 | Isobutyl salicylate | 0.422 | -5.544 |
| 936 | Malathion | 1.874 | -5.486 |
| 231 | Guaifenesin | 1.424 | -5.45 |
| 580 | Chlorhexadol | 2.132 | -5.438 |
| 539 | Tributyrin | 1.72 | -5.436 |
| 786 | Terpinyl propionate | 0.423 | -5.391 |
| 689 | Acetylpyrazine | 0.421 | -5.389 |
| 111 | Ethylpyrazine | 0.422 | -5.383 |
| 710 | Isoamyl salicylate | 2.024 | -5.373 |
| 635 | Chloral Hydrate | 0.422 | -5.359 |
| 795 | Propyl 2-furanacrylate | 2.035 | -5.355 |
| 620 | Allobarbital | 2.296 | -5.285 |
| 274 | Isometheptene Mucate | 0.422 | -5.28 |
| 303 | Mephenesin | 1.476 | -5.278 |
| 790 | Terpinyl isovalerate | 0.423 | -5.267 |
| 497 | Sulfonethylmethane | 2.208 | -5.265 |
| 779 | 3-phenylpropyl acetate | 0.417 | -5.256 |
| 711 | Eugenyl methyl ether | 0.421 | -5.24 |
| 266 | Iodinated Glycerol | 2.086 | -5.231 |
| 834 | Gly-leu | 0.419 | -5.219 |
| 455 | Arecoline Hydrobromide | 0.422 | -5.209 |
| 760 | Phenethyl formate | 0.423 | -5.044 |
| 101 | Famotidine | 1.682 | -5.008 |
| 777 | Terpinyl formate | 0.422 | -4.968 |
| 742 | Geranyl formate | 0.423 | -4.917 |
| 546 | Valpromide | 0.423 | -4.882 |
| 538 | Triacetin | 0.422 | -4.858 |
| 721 | Isobutyl acetate | 0.416 | -4.598 |
| 741 | Geranyl acetate | 0.423 | -4.584 |
| 720 | Ethyl formate | 0.421 | -4.513 |
| 791 | Nerol | 0.423 | -4.317 |
| 782 | Linalyl formate | 0.423 | -4.288 |
| 730 | 3-heptanol | 0.422 | -4.237 |
| 728 | Isobutyl propionate | 0.416 | -4.216 |
| 773 | 6-methyl-5-hepten-2-one | 0.423 | -4.134 |
| 762 | Propyl acetate | 0.412 | -4.097 |
| 736 | Isoamyl acetate | 0.422 | -4.048 |
| 776 | 1-penten-3-ol | 0.42 | -4.014 |

References

Di Pizio, A., L. M. Kruetzfeldt, S. Cheled-Shoval, W. Meyerhof, M. Behrens and M. Y. Niv (2017). Ligand binding modes from low resolution GPCR models and mutagenesis: chicken bitter taste receptor as a test-case. *Sci Rep* 7, 1: 8223.
